# Multi-objective optimization of solar resource allocation in radial distribution systems using a refined slime mold algorithm

**DOI:** 10.1016/j.heliyon.2024.e32205

**Published:** 2024-05-31

**Authors:** Zebin Wang, Yu Li, Guodao Zhang, Xiaotian Pan, Ji Li

**Affiliations:** a Zhejiang College of Security Technology, Wenzhou, 325000, China; b College of Engineering, Ocean University of China, Qingdao, 266100, China; c Electric Power Research Institute of State Grid Xinjiang Electric Power Co. Ltd., Urumqi, Xinjiang, 830011, China; d Institute of Intelligent Media Computing, Hangzhou Dianzi University, Hangzhou, 310018, China

**Keywords:** Distributed generation resources, Voltage stability, Multi-objective optimization, Solar resource allocation, Slime mold algorithm

## Abstract

The integration of distributed generation resources in power systems offers various advantages, such as peak load management and reduced transmission line congestion. However, it also introduces challenges related to voltage stability. This paper presents a novel multi-objective model for optimizing the allocation of solar resources in radial distribution systems. The model aims to achieve an optimal voltage profile, minimize losses, and maximize penetration levels. To address the conflicting nature of these objectives, a refined multi-objective slime mold algorithm (MOSMA) is proposed. This algorithm demonstrates exceptional capabilities in finding Pareto fronts, avoiding local optima, and effectively solving multi-objective problems compared to other optimization methods. Additionally, the corrected social hierarchy method is integrated to enhance performance. The proposed method is evaluated using a standard system under various operational conditions, showing superior results in terms of maintaining an acceptable voltage profile and significantly reducing losses. The study reveals that while losses decrease for penetration levels ranging from low to medium, they start to increase for levels exceeding 100 %. Notably, the proposed method achieves approximately 12 % system efficiency improvement, as measured by the voltage profile, at a penetration level of 300 %. These findings highlight the effectiveness of the proposed method, even at high penetration levels, surpassing other optimization approaches based on the inverse generation distance parameter.

## Highlights

• Novel multi-objective model for efficient solar resource allocation in radial distribution systems.
• Refined multi-objective slime mold algorithm solves conflicting objectives, avoids local optima.
• Integration of corrected social hierarchy method improves algorithm performance.
• Proposed method outperforms in standard system evaluations, maintaining voltage profiles, reducing losses.
• Method excels at high penetration levels, significantly improving system efficiency.

## Nomenclature

*Zbus*: Impedance matrix for a system with *k* buses
*Zij*: Impedance representing the connection between two buses *i* and *j*
*Vi*: Voltage in bus *i*
*Ploss*(*n*): Active power losses in line *n*
*PTotal*_*loss*: Total active power losses for the power system with *k* buses
*VPI*%: Voltage Profile Improvement index
*k*: Number of nodes (buses) in the distribution network
*Vi*: Voltage magnitude of bus *i*
*Li*: Amount of load at the *i*-th node
*F*3,*i*: Objective function related to the power penetration level of photovoltaic sources
*CF*: Capacity factor
*Pinstalled*: Installed capacity of photovoltaic sources
*Cbus*: Corresponding bus capacity
*PDGi*: Production power of the solar unit at bus *i*
*PDG*_*max*: Maximum allowed capacity for each bus
*Vimin*, *Vimax*: Minimum and maximum voltage magnitude of bus *i*
*Gij*,*Bij*: Elements of the admittance matrix
*PGi*, *PLi*, *Vi*, *Vj*, *θij*, *QGi*, *QLi*: Parameters related to active and reactive power, voltage, and angles
*func*: Nonlinear function
*S*: Solution set of parameters
*funci*: Function of *m* input random variables
*μshare*: Maximum allowed distance between any two agents to be considered part of a niche
*popsize*: Population size.
*Max*_*iteration*: Maximum number of iterations
*Xi*: Positions of slime mold
*t*: Current iteration
*W*: Weight of the slime mold
*p*: Parameter for each search portion
*vb*: Parameter ranging from -a to a
*vc*: Vector linearly decreasing from one to zero
*X*: Current location of the slime mold
*XA*,*XB*: Randomly selected slime mold values
*DF*: Best response obtained across all iterations
*r*: Random value in the interval [0,1]
*bF*,*wF*: Optimal and worst values obtained during the current iteration process
*SmellIndex*: Values sorted based on the best response
*x*1,*x*2: Solutions in a multi-objective optimization problem
*Share*(*dij*): Sharing function values for agents in the first front
Crowding distance: Measure used to assess the diversity of solutions in the population
*FDMk*: Normalized membership function for each non-dominated solution
*i*: Index representing the objective function
*μi*: Membership function for the *i*-th objective function
*Nobj*: Number of objective functions

## 1 Introduction

The imperative to address environmental concerns, depleting fossil fuel reserves, and geopolitical energy dependencies has driven a shift towards more efficient fossil energy utilization [1]. Developed countries, recognizing the need to reduce greenhouse gas emissions, particularly CO2, and energy consumption, initially embraced centralized electric power production [2]. While this centralized model offered economic advantages and minimized disturbances, it posed challenges in the face of climate change, leading to significant energy losses and emissions during electricity transmission [3]. Recent research has focused on the inefficiencies of centralized energy networks, highlighting the advantages of distributed generation near consumption areas [4]. Distributed generation, especially from renewable sources like solar, wind, and biomass, has emerged as a sustainable alternative, providing cleaner energy, reducing transmission losses, and enhancing production reserve capacity [5]. Photovoltaic systems, harnessing solar energy with numerous benefits, have garnered significant attention [6]. The concept of photovoltaic penetration level, representing the injected power from these systems into the feeder, has become pivotal in recent studies exploring the impact of high penetration on voltage quality in distribution networks. This transformative shift towards decentralized, sustainable energy sources reflects a broader global commitment to environmental responsibility and energy resilience [[7], [8], [9]]. Various optimization methods have been proposed to determine the optimal allocation of solar systems in distribution networks, considering different objectives such as maximizing penetration level. These objectives can encompass economic goals, power loss reduction, voltage profile improvement, and reactive power considerations. In Ref. [10], a probabilistic hybrid strategy is proposed to determine the allowable penetration of distributed generation resources in distribution networks. The strategy focuses on minimizing losses, ensuring voltage stability, and considering load variations and power factors. It utilizes probabilistic modeling and an enhanced version of the bee algorithm for optimization.

Article [11] presents a method to determine the maximum permissible capacity of distributed generation resources while considering static voltage stability. It evaluates the network structure and load distribution model to determine the penetration of energy sources, considering voltage stability. Article [12] introduces a method to determine the maximum penetration level of distributed generation sources, considering harmonic constraints and protection coordination schemes. The paper emphasizes permissible harmonic distortion and explores the effects on overcurrent relays. A genetic algorithm is utilized for optimization. In article [13], a method is proposed to determine the maximum penetration level of microgrids from the perspective of frequency stability. The strategy combines power system relationships and minimum frequency deviations to analyze distributed generation penetration. Article [14] presents a method to determine the maximum power that distributed generators can inject into each system bus while considering voltage limitations. It utilizes load distribution solutions, matrix operations, and a linearized system model. Article [15] evaluates the impact of increasing the penetration level of distributed generation sources on voltage in the distribution network. It investigates the influence of distributed generation unit size on the maximum allowable penetration level. Article [16] determines the maximum penetration level of distributed generation sources by analyzing voltage and current operating limits. It evaluates the effects of network operating conditions on the penetration level using sensitivity analysis. Article [17] introduces a method for designing active distribution networks considering the effects of distributed generation sources. It investigates the impact of distributed production arrangement on network management and resource capacity. In article [18], a method is presented for optimal voltage regulation in the presence of high-penetration photovoltaic sources. It minimizes solar inverter output interruption using mixed-integer quadratic programming and real-time optimization. Article [19] proposes a coordinated voltage regulation method based on predictive control in the presence of distributed generation sources. It updates voltage sensitivity coefficients in real-time to enhance control efficiency. In the article [20], a voltage control method based on optimal utilization of storage resources is presented. It utilizes the adaptive droop method with the storage system for voltage regulation in the low voltage network. Article [21] focuses on utilizing smart solar inverters for voltage regulation in distribution networks. It incorporates the greedy method to enhance reactive inverter power capability. In article [22], a real-time voltage regulation method is introduced for distribution networks integrating wind power. It determines the control plan considering reactive power, power generators, and traditional voltage controllers. Article [23] proposes a coordinated voltage regulation method based on On-Load Tap Changer (OLTC) and inverters in the presence of high-penetration photovoltaic sources. It addresses voltage fluctuations caused by solar sources and enhances solar production efficiency. Reference [24] focuses on determining the optimal location and capacity of photovoltaic sources in DC networks to reduce emissions from diesel generators. Linear and non-linear integrated integer programming techniques are used for optimization. Reference [25] presents a method for optimizing the arrangement of renewable energy sources, specifically solar, to maximize their impact on power systems. It aims to increase the penetration level and reduce carbon emissions. In Ref. [26], automatic generation models are proposed for reducing losses in distribution networks with distributed generation sources. The models are designed for low-pressure networks in wide service areas. Reference [27] presents a method for estimating the output power and capacity of photovoltaic systems to establish a baseline for demand response. It utilizes the photovoltaic generation-load separation method based on feature extraction. In Ref. [28], a nonlinear hybrid integer programming model is introduced for the optimal allocation of distributed generation resources in distribution networks. The method aims to reduce losses and improve system efficiency. Reference [29] proposes a method for optimal allocation of probabilities for solar and wind systems. It utilizes a multi-objective stochastic optimization method to improve reliability and reduce losses. Reference [30] presents an optimal allocation method for solar power outage resources considering the presence of electric vehicles. It incorporates load uncertainty, solar systems, and electric vehicles in a two-layer optimization model. Reference [31] utilizes the Big Bang algorithm to determine the optimal size of an independent hybrid system comprising wind and solar sources. It optimizes the system to meet demand while minimizing lifetime cost and ensuring reliability. Reference [32] focuses on the optimization and integration of hybrid energy systems, including hydrogen fuel cells. It evaluates various methods and software tools for hybrid systems, emphasizing environmental goals. Reference [33] introduces a method for determining the optimal capacity of solar resources to improve reliability and reduce losses. It utilizes the *Meta*-PSO algorithm for multi-objective optimization. Table 1 provides a comprehensive overview of various papers on distributed generation in distribution networks, highlighting their proposed strategies, focuses, optimization algorithms used, and specific considerations.

**Table 1:** Comparison of papers on distributed generation in distribution networks.

Reference	Proposed Strategy	Focus	Considerations
[10]	Probabilistic hybrid strategy	Minimizing losses, ensuring voltage stability, considering load variations and power factors	Probabilistic modeling, enhanced bee algorithm
[11]	Maximum permissible capacity	Considering static voltage stability	Network structure, load distribution model
[12]	Maximum penetration level	Considering harmonic constraints and protection coordination schemes	Harmonic constraints, protection coordination, genetic algorithm
[13]	Maximum penetration level	From the perspective of frequency stability	Power system relationships, minimum frequency deviations
[14]	Maximum power injection	Considering voltage limitations	Load distribution solutions, matrix operations, linearized system model
[15]	Impact of increasing penetration level	On voltage in the distribution network	Influence of distributed generation unit size on penetration level
[16]	Maximum penetration level	Analyzing voltage and current operating limits	Effects of network operating conditions on penetration level
[17]	Designing active distribution networks	Considering effects of distributed generation sources	Impact of distributed production arrangement on network management and resource capacity
[18]	Optimal voltage regulation	In the presence of high-penetration photovoltaic sources	Minimizing solar inverter output interruption
[19]	Coordinated voltage regulation	Based on predictive control	Control efficiency enhancement
[20]	Voltage control method	Based on optimal utilization of storage resources	Voltage regulation in low voltage network
[21]	Utilizing smart solar inverters	For voltage regulation in distribution networks	Enhancing reactive inverter power capability
[22]	Real-time voltage regulation	Integrating wind power	Control plan determination
[23]	Coordinated voltage regulation	Based on OLTC and inverters	Addressing voltage fluctuations caused by solar sources
[24]	Optimal location and capacity	Of photovoltaic sources in DC networks	Reduction of emissions from diesel generators
[25]	Optimization of renewable energy sources arrangement	Specifically solar	Maximizing impact on power systems, increasing penetration level, reducing carbon emissions
[26]	Automatic generation models	For reducing losses in distribution networks	Designed for low-pressure networks in wide service areas
[27]	Estimation of photovoltaic system output power and capacity	For establishing a baseline for demand response	Utilizes feature extraction
[28]	Nonlinear hybrid integer programming model	For optimal allocation of distributed generation resources	Reduction of losses, improvement of system efficiency
[29]	Optimal allocation of probabilities	For solar and wind systems	Improvement of reliability, reduction of losses
[30]	Optimal allocation method	For solar power outage resources	Load uncertainty, solar systems, electric vehicles in a two-layer optimization model
[31]	Big Bang algorithm	For optimal size of an independent hybrid system comprising wind and solar sources	Meeting demand, minimizing lifetime cost, ensuring reliability
[32]	Optimization and integration of hybrid energy systems	Including hydrogen fuel cells	Evaluation of various methods and software tools, emphasis on environmental goals
[33]	Determining optimal capacity of solar resources	*Meta*-PSO algorithm for multi-objective optimization	Improvement of reliability, reduction of losses

The comprehensive overview of existing literature highlights a well-established body of research on optimizing the allocation of photovoltaic resources. However, a noticeable gap in the literature is identified: the limited attention given to the impact of distributed production resource penetration levels. While previous studies have focused on various objectives, including economic goals, power loss reduction, voltage profile improvement, and reactive power considerations, there is a distinct lack of emphasis on the specific challenges and optimizations related to distributed production resource penetration.

The proposed research aims to address this gap by introducing a novel multi-objective optimization method, namely the slime mold algorithm. This method is presented as a solution to the common problem of getting trapped in local optima, which can impede the effectiveness of traditional optimization approaches like genetics and particle swarming. The incorporation of the corrected social hierarchy method further enhances the efficiency of the proposed multi-objective optimization method. In contrast to the prevailing trend of single- or dual-objective approaches in existing literature, the research stands out by adopting a multi-objective perspective. The key optimization targets are the voltage profile and losses in the distribution system. By determining the optimal capacity of solar resources under high penetration levels, the proposed algorithm seeks to strike a balance between maintaining a stable voltage profile and minimizing losses, considering both technical requirements and economic considerations. This research, therefore, contributes not only by introducing a new and potentially more effective optimization method but also by focusing on a specific aspect that has been underrepresented in previous studies. By addressing the impact of distributed production resource penetration levels, the study aligns with the current environmental and energy challenges, providing insights that can contribute to more sustainable and efficient energy systems. The incorporation of the slime mold algorithm and the corrected social hierarchy method adds innovation to the methodology, making this research a noteworthy contribution to the field of distributed energy resource optimization. The study has the following key contributions.

1. Introduces a novel multi-objective model for optimal solar resource allocation in radial distribution systems, addressing optimization challenges in distributed energy resources.
2. Develops a refined slime mold algorithm tailored for multi-objective optimization, effectively handling conflicting objectives and avoiding local optima.
3. Incorporates the corrected social hierarchy method into the algorithm, enhancing its performance and showcasing an innovative integration of methods.
4. Conducts comprehensive evaluations, demonstrating the proposed algorithm's superior performance in maintaining voltage profiles and reducing losses, serving as a benchmark for comparison.
5. Expands beyond algorithm development by analyzing the impact of penetration levels on system efficiency, providing valuable insights into scalability and effectiveness, particularly at high penetration levels.

In summary, the contributions encompass not only the development of a novel multi-objective model and a refined optimization algorithm but also the integration of innovative methods to enhance performance. The comprehensive evaluations and insights into the impact of penetration levels on system efficiency further strengthen the significance of this research in the field of distributed energy resource optimization. The subsequent sections of the article are organized as follows: Section 2, labeled Methodology, is dedicated to exploring problem modeling and articulating the study's objectives. Section 3, Proposed Algorithm, introduces the developed algorithm designed for energy management optimization. The application of this algorithm is discussed in Section 4. Transitioning to Section 5, Results and Analysis, this part presents the simulation outcomes and numerical analyses. Lastly, Section 6, entitled Conclusions and Future Work, functions as the concluding segment, providing a summary of the findings, drawing conclusions from the study, and proposing potential avenues for future research.

## 2 Mathematical modeling

Within this section, our objective is to elucidate the problem formulation concerning the determination of optimal capacities for photovoltaic systems across all buses interconnected in the distribution network. The optimal resolution of the multi-objective optimization problem will encompass factors such as the penetration level of solar generation sources, enhancement of the voltage profile, and mitigation of active power losses [[34], [35], [36], [37], [38], [39], [40], [41], [42], [43], [44], [45]].

### 2.1 load distribution analysis

The objective of load spreading is to ascertain the voltage size and angle across all buses during operation. The relationship of load distribution is articulated in terms of the impedance or admittance matrix. Typically, the impedance matrix for a system with k buses is denoted as Equation (1) [45].

(1) $$Z_{\text{bus}}=\left[\begin{array}{ccc} Z_{1} & \cdots & -Z_{1k}\\ \vdots & \ddots & \vdots \\ -Z_{1k} & \cdots & Z_{\text{kk}} \end{array}\right]$$

**Fig. 1:**
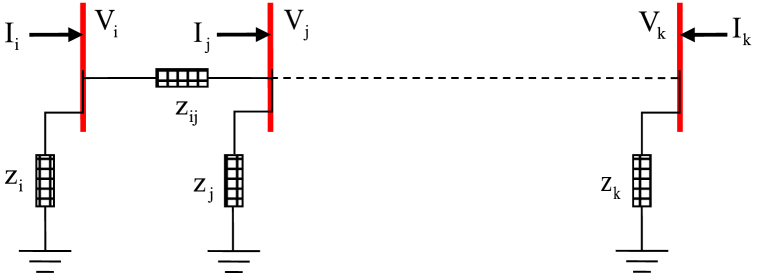
Sample distribution network for i-k buses.

All branches with their impedance values are shown in Fig. 1. The impedance Z_ij_ represents the connection between two buses i and j. The voltage in each bus i is calculated by Equation (2):

(2) $$V_{i}=\sum_{i=1}^{k}Z_{\text{ij}}I_{i}$$

### 2.2 power losses

**Fig. 2:**
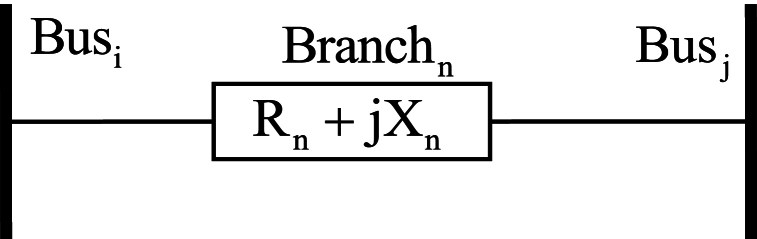
Part of the distribution network.

A part of the distribution network is shown in Fig. 2, where two buses i and j are connected through a line, and line n is represented by reactance X(n) and resistance R(n). Active power losse in line n are represented by Ploss(n) and given by Equation (3) [33,45]:

(3) $$P_{loss}\left(n\right)=R\left(n\right).\left(\left(P^{2}+Q^{2}\right)/{\left|V_{j}\right|}^{2}\right)$$

The total active power losses for the power system including k buses can be obtained by summing up the losses of all its lines with Equation (4) [33]:

(4) $$P_{Total\_loss}=\sum_{n=1}^{k}P_{loss}\left(n\right)$$

Equation (5) characterizes the active power output of a photovoltaic power plant within a distribution network, and it is formulated based on established relationships cited in Refs. [33,34]:

(5) $$P_{pv}=\sqrt{\frac{V_{i}^{2}}{R_{i}}P_{pvloss}-\left(P_{i}^{2}+Q_{i}^{2}\right)+(Q_{pv}^{2}-2P_{i}P_{pv}-2Q_{i}Q_{pv}(\frac{G}{L}})$$

In this equation, P_pv_ denotes the real power supplied by the photovoltaic system, and Q_pv_ represents the reactive power injected by the photovoltaics. The variables G and L stand for the distance from the photovoltaic source in kilometers and the total length of the feeder from the source to bus i, respectively. This equation serves as a crucial element in modeling the active power contribution of photovoltaic sources in the distribution network, taking into account various electrical parameters and system characteristics. Therefore, the reduction of total power loss is defined by Equation (6) as the difference in power loss before and after photovoltaic installation [34,45]:

(6) $$P_{NLR}=\frac{R_{i}}{V_{i}^{2}}\left(P_{PV}^{2}+Q_{PV}^{2}-2P_{i}P_{pv}-2Q_{i}Q_{pv}\right)\left(\frac{G}{L}\right)$$

A positive value of *P*_*NLR*_ indicates a decrease in system losses due to the presence of photovoltaic sources, while a negative value indicates an increase in losses.

### 2.3 improving the voltage profile

The second objective function of the problem aims to improve the voltage profile. For this purpose, the voltage profile improvement (VPI) index is used, which is calculated as follows. Equation (7) describes the criteria for improving the VPI index. In this research, this parameter is used to assess the improvement of the voltage profile after the allocation of photovoltaic sources [35,45]:

(7) $$VPI\%=\left(\frac{{\left(\sum_{i=1}^{k}V_{i}L_{i}\right)}_{PV}-{\left(\sum_{i=1}^{k}V_{i}L_{i}\right)}_{0}}{{\left(\sum_{i=1}^{k}V_{i}L_{i}\right)}_{0}}\right)\times 100$$

In this equation, k is the number of nodes (buses) in the distribution network, V_i_ is the voltage of each node, and L_i_ is the amount of load at the ith node. Subscript 0 represents the state before photovoltaic implementation, and PV subscript refers to the state after the installation of photovoltaic sources.

### 2.4 photovoltaic power level

The third objective function, which is related to the power penetration level of photovoltaic sources, is given by the following equation. The goal is to maximize the penetration level of the source, although at high penetration levels, it can lead to a power imbalance between demand and production and given by Equation (8) [[33], [34], [35]]:

(8) $$F_{3,i}=CF\times P_{installed}/C_{bus}$$

In the above equation, CF is the capacity factor, P_installed_ is the amount of installed capacity, and C_bus_ is the corresponding bus capacity. This quantity represents the ratio of the injected energy from distributed generation to the network to the capacity of the corresponding feeder or bus.

### 2.5 constraints of the problem

The constraints of the distribution system design problem in the presence of distributed generations are calculated by Equation (9) [33]:

(9) $$0\leq P_{DGi}\leq P_{iDG}^{\max},V_{i}^{\min}\leq V_{i}\leq V_{i}^{\max}$$

In this relation, $P_{iDG}^{\max}$ represents the maximum allowed capacity in each bus, and $P_{DGi}$ is the production power of the solar unit. Another constraint that should be considered is the load distribution constraint, which is expressed by Equations (10), (11) [34]:

(10) $$P_{Gi}-P_{Li}-V_{i}\sum_{j\in Ni}V_{j}[G_{ij}\;\cos\left(\theta_{ij}\right)+B_{ij}\;\sin\left(\theta_{ij}\right))=0$$

(11) $$Q_{Gi}-Q_{Li}-V_{i}\sum_{j\in Ni}V_{j}[G_{ij}\;\sin\left(\theta_{ij}\right)-B_{ij}\;\cos\left(\theta_{ij}\right))=0$$

In these equations, $P_{Gi}$ represents the active power generated at bus i, $P_{Li}$ is the active power of the load at bus i, $V_{i}$ is the voltage magnitude of bus i, $V_{j}$ is the voltage magnitude of bus j, $\theta_{ij}$ is the angle between bus i and j, $Q_{Gi}$ is the reactive power at bus i, and $Q_{Li}$ is the reactive power at bus i.

### 2.6 uncertainty characterization

Considering the inherent unpredictability of wind speed and the uncontrollable coefficients linked to load demands, it becomes crucial to incorporate these variables into a probabilistic framework. In the realm of system management, the system operator needs to define the distribution functions of output variables, taking into account the distribution functions of input random uncertainties. For clarity, we can represent a nonlinear function as "func" [9].

(12) S=func(z)

In Equation (12), elucidating the solution set of parameters, denoted as S, requires a precise depiction of the uncertainties associated with both wind speed and load demand. To achieve this, the 2 m point estimated method is applied to compute the moments of Si. This function, represented as "funci," depends on m input random variables and is defined by Equation (13) [9].

(13) $$S_{i}=func_{i}\left(z_{1},z_{2},\ldots ,z_{m}\right)$$

In the usual practice of the 2 m point estimated method, the probability density function fzl for variable zl is commonly estimated through two points. This estimation involves utilizing the first three central moments obtained from statistical data, specifically the mean (μzl), variance (σzl), and skewness (λzl,3) coefficients.

## 3 Proposed multi-objective optimization solution algorithm

In the rapidly evolving landscape of energy systems, active distribution grids have emerged as key players in facilitating the integration of renewable energy sources and enhancing grid resilience [36]. Multi-objective optimization, as applied to active distribution grids, plays a pivotal role in addressing the diverse and often conflicting objectives inherent in such complex systems. This approach involves simultaneously optimizing multiple criteria, such as minimizing power losses, improving voltage profiles, and maximizing renewable energy penetration. The formulation of these multi-objective optimization problems is a critical aspect that shapes the decision-making processes in energy management [37]. Researchers have explored various optimization algorithms and frameworks to achieve efficient and sustainable solutions. These formulations not only consider technical aspects like grid reliability and power quality but also incorporate economic factors and environmental sustainability [38].

The Slime Mold Algorithm (SMA) stands out as an innovative metaheuristic inspired by the dynamic behavior of slime molds in nature. Distinguishing itself with unique features and a mathematical model employing adaptive weights derived from bio-oscillators, SMA optimizes the path-finding process for food binding, showcasing exceptional discovery capabilities [39]. In contrast to other metaheuristic algorithms such as GA and PSO, SMA displays faster convergence and attains desired solutions within a shorter computational time frame. Subsequent sections delve into elucidating the mathematical foundations that underlie the functionality of this algorithm.

### 3.1 mathematical model of SMA

Slime molds exhibit a navigation mechanism towards food sources by tracing airborne chemical signals. To encapsulate this behavior in mathematical terms, we introduce Equation (14) [39]:

(14) $$\vec{X}\left(t+1\right)=\left\{ \begin{array}{l} \vec{X_{b}}\left(t\right)+\vec{vb}.\left(\vec{W}.\vec{X_{A}}\left(t\right)-\vec{X_{B}}\left(t\right)\right),r<p\\ \vec{vc.}\vec{X}\left(t\right),r\geq p \end{array}\right.$$

In this context, vb is a parameter spanning from -a to a, while vc is a vector that linearly diminishes from one to zero. The variable t signifies the current iteration, Xb indicates the location with the highest prevailing odor concentration, and X denotes the present location of the slime mold. XA and XB denote randomly chosen values associated with the slime mold, and W signifies the weight of the slime mold. Furthermore, the calculation for the relationship of p is determined by Equation (15) [39]:

(15) p=tanh|S(i)−DF|

In this context, where i takes values from 1 to n, and S(i) represents the fitness coefficient of X, DF represents the best response acquired across all iterations [39]. The function range for vb, and the correlation between a and W, are delineated by Equations (16), (17), (18), (19) [39]:

(16) $$\vec{vb}=\left[-a,a\right]$$

(17) $$a=\arctan\;h\left(-\left(\frac{t}{\max\_t}\right)+1\right)$$

(18) $$\vec{W\left(SmellIndex\right)}=\left\{ \begin{array}{l} 1+r.\log\left(\frac{bF-S\left(i\right)}{bF-wF}\right)+1),condition\\ 1-r.\log\left(\frac{bF-S\left(i\right)}{bF-wF}\right)+1),others \end{array}\right.$$

(19) SmellIndex=sort(S)

Within this framework, the condition emphasizes the primary significance of S(i) within the population. The variable r takes on a random value within the range [0,1], bF denotes the optimal coefficient achieved in the ongoing iteration process, wF signifies the worst value obtained during the current iteration process, and SmellIndex represents values sorted according to the best response. Table 2 [39] provides the pseudo code for the SMA algorithm.

**Table 2:** SMA pseudo code.

**Algorithm 1** Pseudo-code of SMA
**Initialize the parameters popsize, *Max_iteration;*** **Initialize the positions of slime mold *X***_***i***_**(*i*=1, 2, …, n);** While **(*t* < *Max_iteration*)** **Calculate the fitness of all slime mold;** **update *bestFitness*, *X***_***b***_ **Calculate the *W*;** For **each search portion** **Update p, vb, vc** End **For;** ***t*=*t* + 1;** End While; Return ***bestFitness*, *X***_***b***_**;**

here are three formulations from Equations (20), (21), (22) for a modified SMA algorithm incorporating local search and dynamic operators:

**Local Search Formulation:** The local search operation introduces a small perturbation to the current solution *Xi* based on the gradient of the objective function:

(20) $$Xi^{\prime }=Xi+\delta ∙\nabla f\left(Xi\right)$$

where *δ* is a positive step size, and ∇*f*(*Xi*) represents the gradient of the objective function at *Xi*.

**Dynamic Operator for Parameter Adaptation:** The dynamic operator adapts algorithmic parameters based on the evolving landscape. Let *P*(*Xi*) denote a dynamic parameter, and *α* be the adaptation rate:

(21) P(Xi+1)=P(Xi)+α∙∇P(Xi)

where ∇*P*(*Xi*) is the gradient of the parameter *P* at *Xi*.

**Combined Local Search and Dynamic Operator:** Combining the local search and dynamic operator, the modified SMA algorithm can be expressed as:

(22) $$Xi^{\prime }=Xi+\delta ∙\nabla f\left(Xi\right)+\beta ∙\nabla P\left(Xi\right)$$

where *β* represents a weighting factor between the local search and dynamic operator components. These formulations aim to enhance the SMA algorithm's capability to explore and exploit the search space effectively through local refinement and adaptive parameter adjustments.

### 3.2 non-dominated sort (NDS)

The NDS technique is used to find multiple trade-off solutions by assigning Pareto ranks to solutions and promoting diversity while aiming for the Pareto optimal front [40]. In the realm of multi-objective optimization problems, the relationship between two solutions, ×1 and x2, can be categorized into one of two scenarios: either one solution dominates the other, or there is no dominance. The conditions for ×1 dominating ×2 are encapsulated in Equations (23), (24), where both conditions must be met simultaneously [40]:

(23) $$\forall i\in \left\{1,2,\ldots N_{obj}\right\}:f_{i}\left(x_{1}\right)\leq f_{i}\left(x_{2}\right)$$

(24) $$\forall j\in \left\{1,2,\ldots N_{obj}\right\}:f_{j}\left(x_{1}\right)\leq f_{j}\left(x_{2}\right)$$

If ×1's objective values are less than or equal to ×2's values and there exists at least one objective where ×1's value is strictly less than x2's value, ×1 dominates x2. A non-dominated solution is not dominated by any other solution. After ranking the population, individuals in the first non-dominated front with rank 1 receive a high fitness value. To maintain diversity, a sharing strategy assigns lower fitness values to individuals in subsequent fronts based on the minimum shared fitness value of the previous front. This process continues until shared fitness values are determined for all fronts. Based on Equations (25), (26), the sharing function values (Share(dij)) is given [[36], [37], [38], [39]]:

(25) $$\text{Share}\left(d_{ij}\right)=\left\{ \begin{array}{l} 1-{\left(\frac{d_{ij}}{\mu_{share}}\right)}^{2},ifd_{ij}<\mu_{share}\\ 0,otherwise \end{array}\right.$$

(26) $$d_{ij}=\sqrt{\sum_{a=1}^{P_{1}}{\left(\frac{x_{s}^{i}-x_{s}^{j}}{x_{s}^{\max}-x_{s}^{\min}}\right)}^{2}}$$

where, p1 signifies the overall count of decision variables, xs indicates the value of the sth decision variable, and i and j denote distinct agent numbers. The parameter μshare represents the maximum permissible distance between any two agents to qualify as part of a niche. The determination of the niche count for the entire population (N) is computed by Equation (27):

(27) $$Nichecount_{i}=\sum_{j=1}^{N}\text{Share}\left(d_{ij}\right)$$

### 3.3 crowding distance

**Fig. 3:**
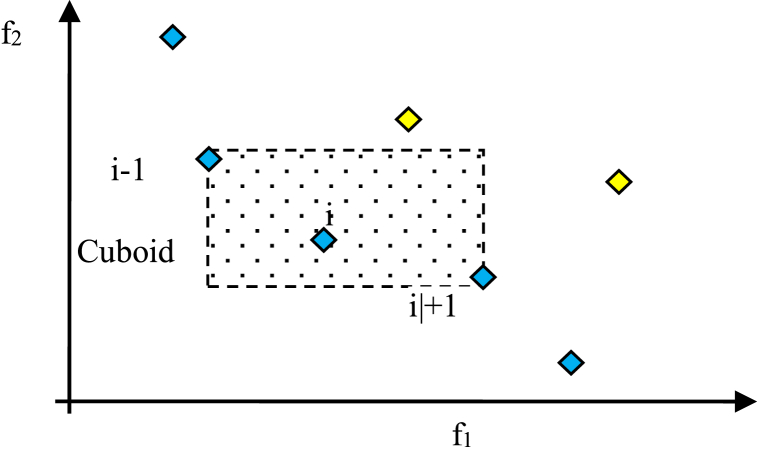
illustrates the calculation of the crowding distance.

The crowding distance is a measure used in multi-objective optimization algorithms to assess the diversity of solutions in the population. It is assigned to individuals after the non-dominated sort. (as depicted in Fig. 3). The crowding distance reflects the density of solutions in the objective space and helps maintain a well-distributed set of solutions along the Pareto front. To calculate the crowding distance for an individual, we consider its neighboring solutions in the objective space. The crowding distance measures the extent to which the objective space around the individual is unoccupied by other solutions. It is typically calculated by estimating the perimeter or volume of a shape formed by the neighboring solutions.

### 3.4 fuzzy selection

In multi-objective optimization, the proposed algorithm employs fuzzy-based mechanisms and fitness sharing to select the best compromise solution from the Pareto front. Fuzzy set theory is used to efficiently derive a candidate Pareto optimal solution, and linear membership functions are used for each objective function and given by Equations (28), (29) [40]:

(28) $$\mu_{i}=\frac{f_{i}^{\max}-f_{i}}{f_{i}^{\max}-f_{i}^{\min}}$$

(29) $$FDM_{i}=\left\{ \begin{array}{l} 0\\ \mu_{i}\\ 1 \end{array}\right.\;\begin{array}{l} \mu_{i}\leq 0\\ 0<\mu_{i}<1\\ \mu_{i}\geq 1 \end{array}$$

**Fig. 4:**
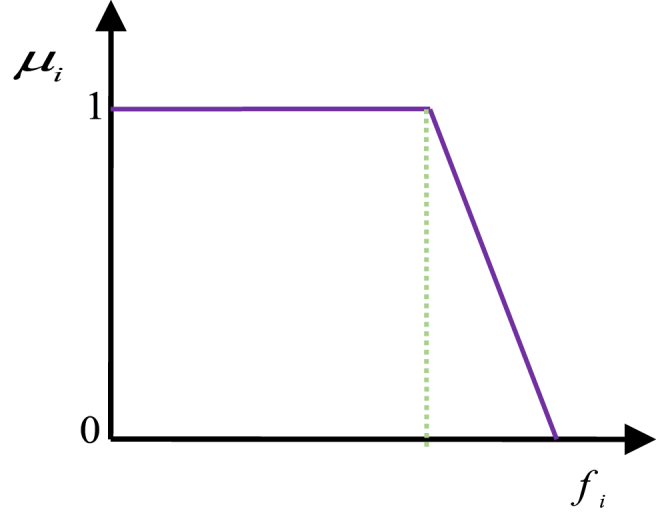
the membership function.

The normalized membership function FDMk for each non-dominated solution k is calculated by Equation (30):

(30) $$FDM^{k}=\left[\frac{\sum_{i=1}^{N_{\text{obj}}}FDM_{i}^{k}}{\sum_{j=1}^{M}\sum_{i=1}^{N_{\text{obj}}}FDM_{i}^{j}}\right]$$

where $f_{i}^{\min}$ and $f_{i}^{\max}$ represent the minimum and maximum values of the ith objective function, respectively. Fig. 4 provides a visual representation of a typical shape of the membership function.

## 4 Application of the algorithm

The multi-objective SMA method is employed to address the problem of optimizing the allocation of photovoltaic resources. The goal is to determine the optimal capacity and location of photovoltaic sources to simultaneously minimize all three objective functions. The proposed procedure consists of the following steps:

Initialization: The population is initialized using the SMA method, considering parameters such as the number of photovoltaic sources, maximum repetitions, and population size.

Definition of Design Variables: Each photovoltaic source requires an optimal capacity. The number of optimization variables is determined using Equation (31):

(31) $$V_{ar}=2N_{pv}$$

The first bus in the distribution network is designated as the slack bus and cannot be selected as the position for a photovoltaic source.

Setting Lower and Upper Limits: The lower limit (Llim(f)) of Var is defined for the second bus onwards, along with the minimum capacity (cmin), depending on the photovoltaic penetration level. Similarly, the upper limit (Ulim(f)) includes the last bus in the distribution network and the maximum capacity (cmax) based on the photovoltaic penetration surface.

Population Formation: A population matrix is created, consisting of the capacity (C) and position (S) variables for each solar power plant. The capacity and position information is included in the population matrix.

System Evaluation: The distribution system information is implemented and evaluated by assessing load losses and other relevant parameters.

Multi-objective Optimization: The multi-objective SMA algorithm is executed, considering the three defined objectives: minimizing losses, improving the voltage profile, and achieving the desired penetration level. During the simulation, the level of photovoltaic penetration is assessed and treated as a constraint to ensure an appropriate level of penetration.

By following these steps, the multi-objective SMA procedure facilitates the optimal allocation of photovoltaic resources while considering multiple objectives and constraints.

## 5 Simulation results and analyses

The simulations in this section were conducted utilizing MATLAB software, specifically the 2020 version. The computational tasks were performed on a personal computer featuring robust hardware specifications, including a 7-core 2.53 GHz processor, 8 GB RAM, 12 MB cache memory, and a 512 GB internal hard drive. These hardware configurations provided an optimal environment for running the simulation experiments and obtaining accurate results.

### 5.1 Test system

**Fig. 5:**
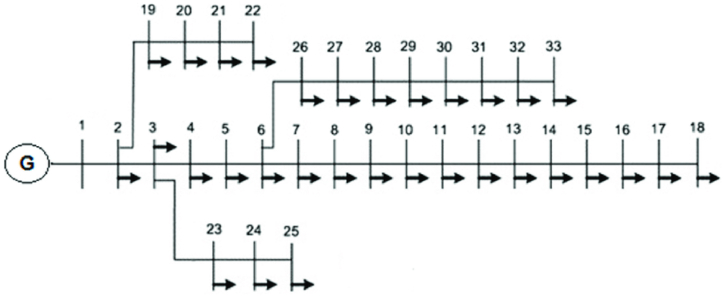
Single line diagram of 33 bus network.

To assess the performance of the proposed method, the distribution network consisting of 33 standard buses is selected. Fig. 5 illustrates the single-line diagram of the 33-bus distribution network [41]. The system under consideration has a total demand of 3715 kW and 2300 kW. The network information relevant to the optimal allocation analysis is presented in Appendix.

### 5.2 simulation results

This section presents an analysis of the results obtained from implementing the proposed method for allocating photovoltaic resources. Various definitions exist for the penetration level of photovoltaic sources [41,42]. In this study, the photovoltaic penetration level is defined as the ratio of the maximum photovoltaic power to the maximum apparent power of the load [43]. Penetration is expressed as a percentage of the total capacity of the photovoltaic system. The scenarios considered include penetration levels of 100 % and 300 % of the annual peak load [44]. The proposed method for achieving optimal allocation of the photovoltaic system is applied to the 33-bus test network in this section. The simulation results validate the effectiveness of the proposed method in reducing losses and improving the voltage profile. The capacities of the photovoltaic systems range from 2.005 to 3.107 MW in different cases. The positions and capacities obtained are presented in Table 3. Table 3 provides a comprehensive assessment of the proposed method's performance in the context of various configurations involving photovoltaic (PV) systems. As the number of PVs increases from 2 to 5, the connected bases and PV system capacities dynamically adjust. Notably, for the case with 2 PVs, connected to buses 6 and 18, the proposed method achieves a PV system capacity range of 0.98–1.02 MW. This leads to an average voltage of 0.98931 pu and losses of 0.07945 MW, showcasing its ability to optimize within this setting. As the complexity increases with 3, 4, and 5 PVs, distributed across different buses, the proposed method consistently demonstrates effective optimization, minimizing losses while maintaining desired voltage profiles. These results underscore the adaptability and efficiency of the proposed method in addressing the challenges posed by the integration of varying numbers of PV systems in distribution networks. Overall, the simulation results demonstrate the effectiveness of the proposed method in optimizing the allocation of photovoltaic resources, reducing losses, and improving the voltage profile.

**Table 3:** Performance of the proposed method for the presence of photovoltaic systems.

losses (MW)	average voltage (pu)	Photovoltaic system capacity (MW)	Connected basses	Number of PVs
0.07945	0.98931	0.98, 1.02	6,18	2
0.06512	0.98734	0.74; 1.06; 1.09	16,18,30	3
0.05847	0.98960	0.56, 0.85, 0.95, 0.78	6,16,28,16	4
0.05439	0.99031	1.53; 0.33; 0.33; 0.35; 0.36	6,18,28,16,31	5

Using the Mat Power set, an analysis was conducted on a 33-bus distribution network without photovoltaic sources. Subsequently, photovoltaic systems were integrated into the network, and the effects of varying penetration levels of photovoltaic sources up to 300 % were analyzed. In this analysis, the capacity of the photovoltaic system was determined for each bus based on the load's apparent power.

### 5.3 Examining the effect of penetration level on the voltage profile

This section investigates the impact of high penetration levels on the voltage profile. The installation of distributed generation sources in a power system leads to voltage increases in the distribution network due to changes in load distribution [45]. When photovoltaic power output experiences a significant increase due to sudden changes in solar radiation, overvoltage may occur in the distribution feeders. Overvoltage can damage equipment and cause malfunctions. In distribution networks with high photovoltaic system penetration, overvoltage is a common concern that must be addressed to ensure reliable and safe system operation. Additionally, the maximum penetration level should be assessed considering overvoltage issues as constraints. Solar power plants inject only active power into the grid, which can alter the distribution of reactive power in the system. Consequently, nearby bus voltages increase due to the lack of reactive power. Normally, the acceptable range for normal operating voltages is between 0.05 and 1.05 per unit.

**Fig. 6:**
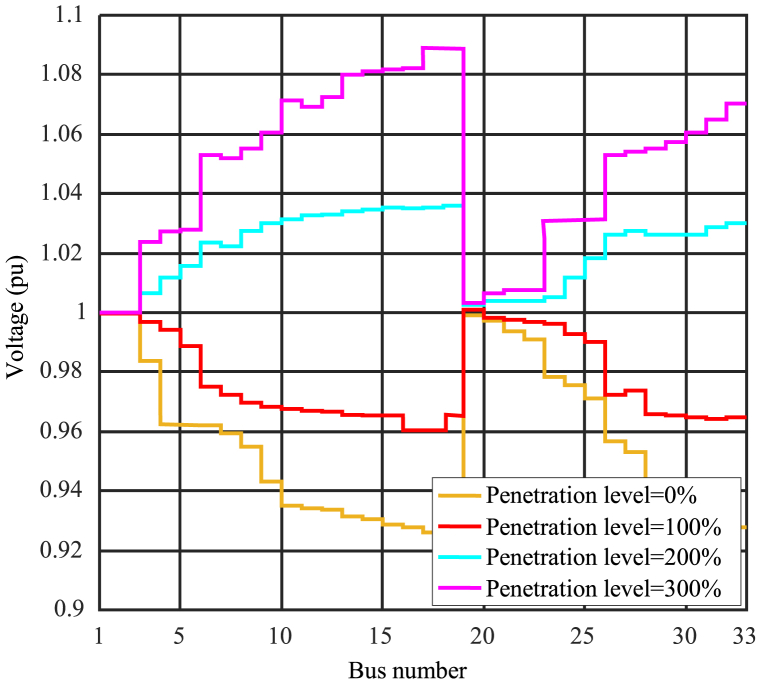
Effect of penetration level of photovoltaic sources on voltage profile.

Fig. 6 illustrates the effects of high photovoltaic penetration levels on the voltage profile of the 33-bus system. The voltage profile remains within the acceptable range for buses 1 to 8 and buses 18 to 27. However, in the weaker buses of the test system, specifically buses 9 to 19 and 28 to 33, the voltages are outside the acceptable range. The maximum voltage at bus 17 for a penetration level of 300 % reaches 1.088 per unit, making it the weakest bus in the network. As depicted in the figure, penetration levels of 200 % and 100 % are more suitable compared to other values in terms of voltage profile. In the 300 % state, the voltage increase is excessively high, creating an unfavorable situation that may require production to be halted until conditions return to a proper state. Similarly, the voltage drop is significant in the case of zero percent penetration.

### 5.4 examining the impact of penetration level on losses

**Fig. 7:**
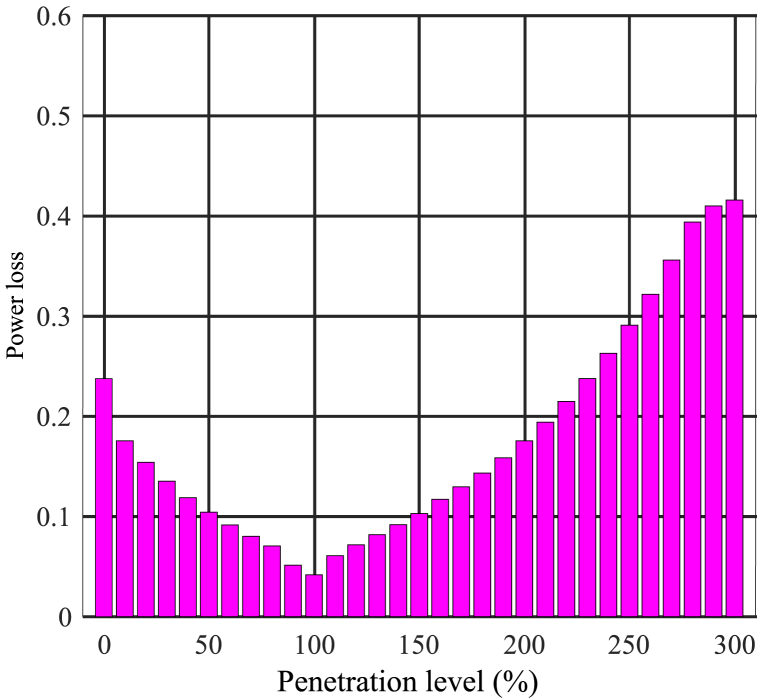
Power losses at different penetration levels.

Typically, as the installed capacity of distributed generation systems gets closer to the load, total losses decrease. However, research indicates that losses tend to increase with higher penetration levels [45]. Fig. 7 illustrates the effect of penetration level on losses in a 33-bus network. It demonstrates that losses are minimized when the output is located near the load. For low to moderate penetration levels, losses are expected to decrease to a minimum value. However, as penetration levels exceed 100 %, losses start to increase. Specifically, increasing the penetration level to 300 % leads to an increase in losses from 0.23 to 0.49 MW. The optimal situation occurs at a penetration level of 100 %, and beyond that, there is an increase in losses, requiring adjustments to the photovoltaic output.

### 5.5 optimal positioning and capacity for high photovoltaic penetration levels

**Fig. 8:**
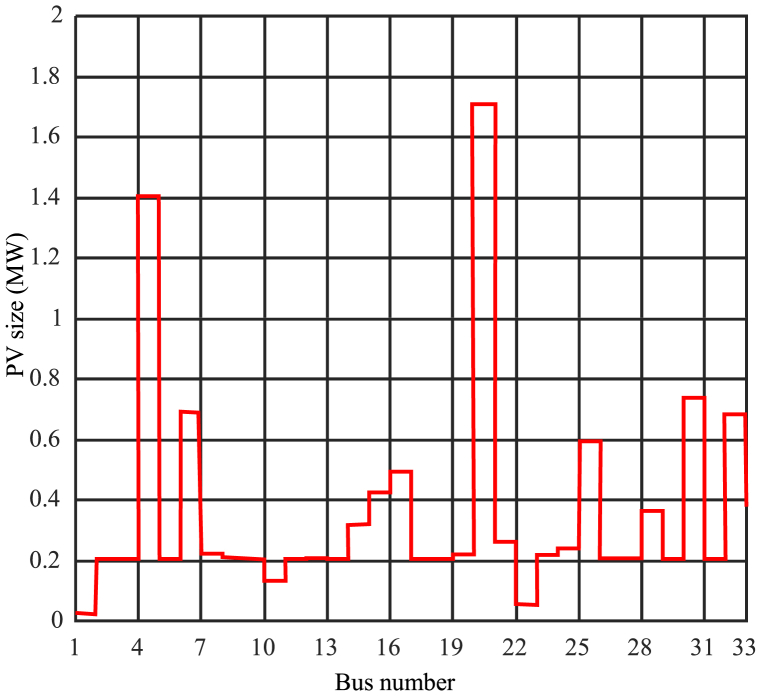
Optimization output for optimal allocation at 300 % penetration level.

High penetration of photovoltaic systems poses challenges for the distribution system. Previously, resource capacity was determined based on the apparent power of the load at each bus, resulting in voltage increases. To address the challenges associated with increased voltage, it is necessary to find the optimal capacity for each bus. In this study, the SMA was utilized to determine the optimal position and capacity, considering 64 variables for location and capacity. Fig. 8 presents the results at different penetration levels. The optimal solution should satisfy the objectives of improving the voltage profile and reducing losses. The assumed scenario involves installing photovoltaics in all buses, with 64 variables defining the position and capacity. A penetration level of 300 % was considered, with a total power of 11.234 MW. The optimization equations in the SMA multi-objective optimizer aim to minimize active losses while considering voltage profile improvement.

The results indicate that the highest capacity is located at the initial buses of the network, specifically buses 2 and 3, with a capacity of 1.88 MW. For lower penetration levels, the terminal buses are selected for installing photovoltaic system capacity to enhance the voltage profile. It is concluded that high penetration rates result in overvoltage in the network. To maintain the voltage profile, the photovoltaic system's capacity should be installed at the primary buses of the network. The maximum and minimum capacities are found to be 1.88 MW and 0.15 MW, respectively.

**Fig. 9:**
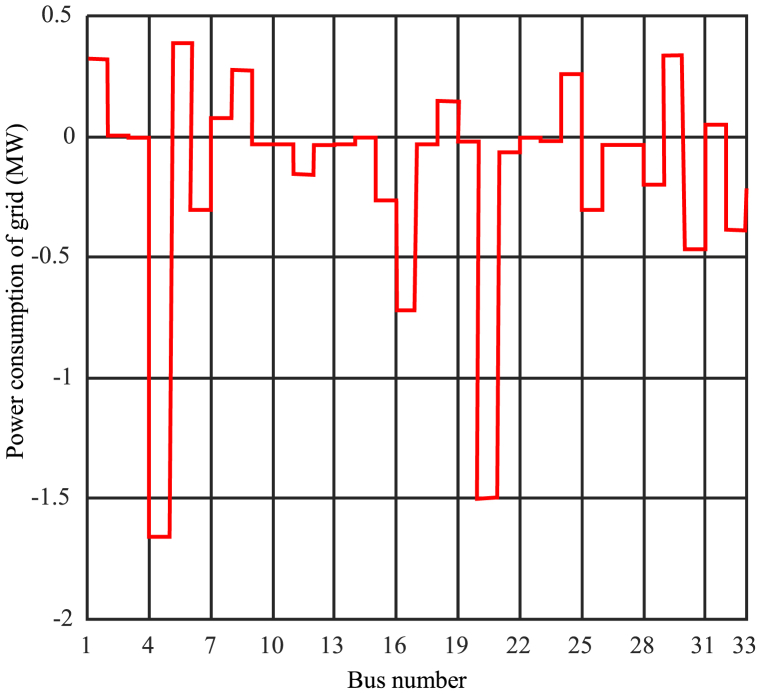
Photovoltaic capacity for network analysis mode under optimization with Cmax = 2 and Cmin = 0.15.

**Fig. 10:**
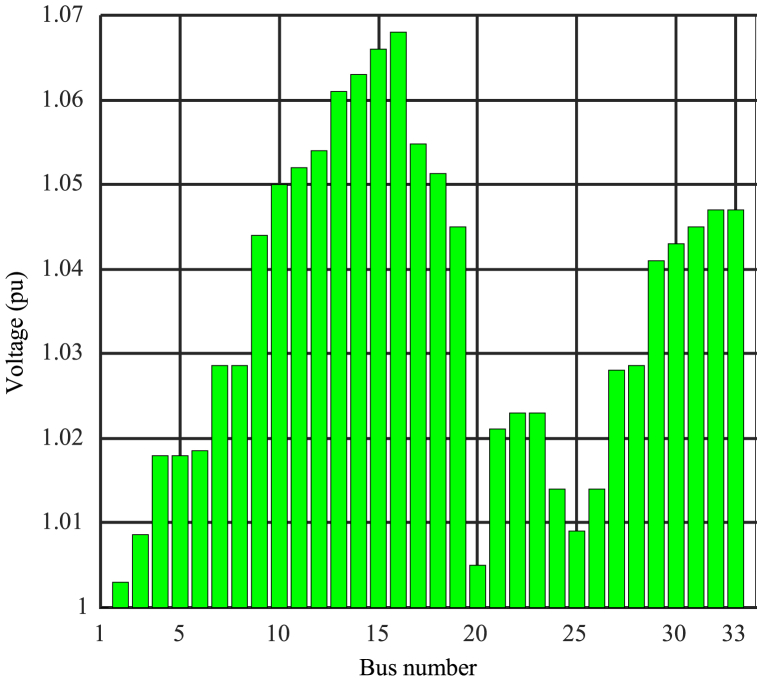
Network voltage analysis mode under optimization with Cmax = 2 and Cmin = 0.15.

**Fig. 11:**
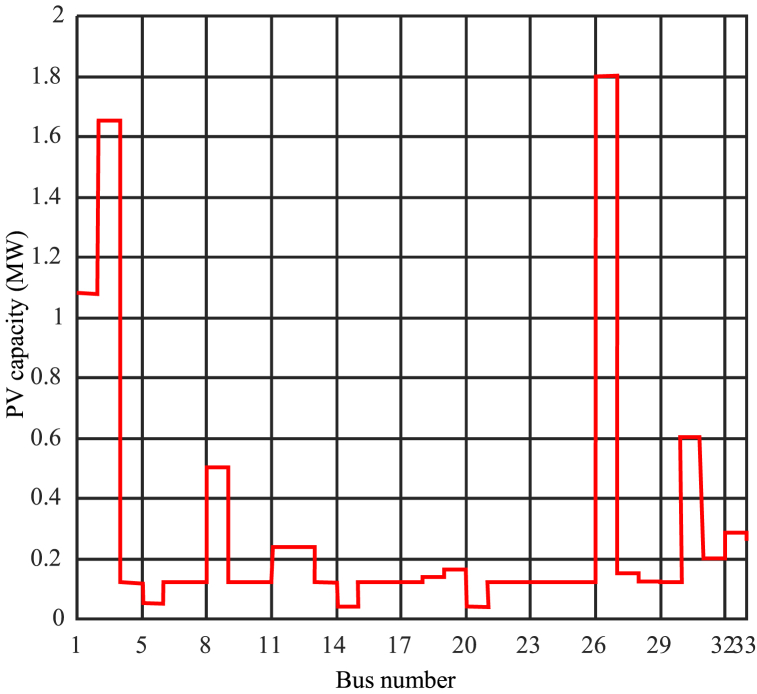
Per-unit voltage for network analysis mode under optimization with Cmax = 2 and Cmin = 0.15.

Determining the optimal penetration level is a complex task that requires precise adjustment of Cmin and Cmax. Based on the results and simulations, selecting Cmax = 2 can be desirable to achieve different objectives and higher penetration levels. Fig. 9 displays the results of network analysis under optimization with Cmax = 2 and Cmin = 0.15. It depicts the schematic representation of photovoltaic capacity in different buses. Furthermore, Fig. 10 illustrates the power consumption from the network in megawatts for different buses, and Fig. 11 shows the per-unit voltage in different buses for this mode.

**Fig. 12:**
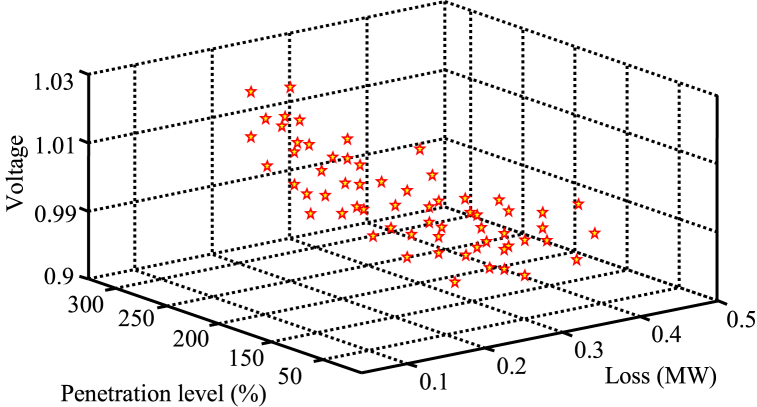
Pareto fronts for solving the three-objective problem.

In this section, the researchers discussed the three-objective solution to the problem, which includes penetration level, voltage profile, and losses. Fig. 12 displays the Pareto fronts obtained from solving the three-objective problem using the corrected SMA method. The figure presents the average value of the actual voltage for f voltage, making the improvement of the voltage profile and its proximity to the per-unit value more comprehensible. Additionally, the voltage at the output of the target function represents the average voltage of all buses. The majority of the response set is concentrated around a penetration level of approximately 100 %, while some responses can be found at higher or lower penetration levels. Most responses fall within the 100 % penetration level range, and the values of losses and voltage profile are within acceptable limits.

In this section, the proposed multi-objective SMA (MOSMA) method is compared with the MOPSO and NSGA-II methods in terms of performance parameters for multi-objective methods. Table 4 presents the comparison of the proposed method with these methods using the parameters introduced in the third chapter, namely IGD and SP. The results indicate that the proposed MOSMA method has the potential to achieve better results than the other two methods in terms of the IGD statistical parameter. The MOSMA method demonstrates higher convergence and accuracy compared to the other two methods, resulting in superior convergence. Additionally, the SP and MS parameters show acceptable performance compared to the other two methods.

**Table 4:** Comparison of the proposed method with MOPSO and NSGA-II methods.

NSGA-II	MOPSO	MOSMA	
0.498	0.352	0.201	Average IGD
0.199	0.203	0.192	Standard deviation of IGD
0.654	0.614	0.528	The worst IGD
0.038	0.048	0.115	The best IGD
0.007	0.006	0.013	Average SP
0.002	0.002	0.001	Standard deviation of SP
0.007	0.007	0.012	The worst SP
0.005	0.005	0.005	Best SP
0.941	0.976	0.973	Average MS
0.005	0.025	0.002	MS standard deviation
0.914	0.913	0.901	The worst MS
0.952	1.015	1.011	The best MS

To identify optimal adjustment coefficients in the proposed algorithm, diverse tests were conducted using various test functions. The results indicated that the optimal configuration involves an initial population size of 60, σ (sigma) set to 0.3, λ (lambda) at N/2, and the program executed for 300 repetitions. In this study, we compare our proposed method with several established multi-objective algorithms, namely NSGA-II, NPGA, SPEA, and MOPSO. Evaluating the performance of multi-objective algorithms is inherently complex, often involving the examination of Pareto front quality. Introducing the C-metric, denoted by equation (32) below, provides a systematic way to compare the solutions between two sets, S1 and S2 [46]:

(32) $$C\left(S_{1},S_{2}\right)=\frac{\left|\left\{a_{2}\in S_{2},\exists a_{1}\in S_{1}:a_{1}≺a_{2}\right\}\right|}{\left|S_{2}\right|}$$

If C(S_1_,S_2_) = 1 then all the answers in set S2 overcome the answers in S1. If C(S_1_,S_2_) = 0 then none of the solutions of S2 can be covered by the set S1. Both pairs C(S_1_,S_2_) and C(S_2_,S_1_) should be checked in comparison until the C-metric has symmetric information. Table 5 shows the result of comparison between five algorithms based on C-metric criteria, where B1, B2, …, B3 refer to MOSMA, NSGA and MOPSO.

**Table 5:** Comparison of different algorithms based on C-metric criteria.

	C(B_1_,B_2_)	C(B_2_,B_1_)	C(B_1_,B_3_)	C(B_3_,B_1_)
Best	0.765	0.0038	0.564	0.0342
Average	0.662	0.0012	0.497	0.0202
Std	0.0023	0.0002	0.0031	0.0002

In the comparison between MOSMO and NSGA, MOPSO consistently outperforms with 76.5 % best coverage and 66.2 % average coverage. The standard deviation for MOSMA remains low at 0.23 %. When compared to MOPSO, MOSMA continues its superior performance, with 56.4 % best coverage and 49.7 % average coverage, accompanied by a stable standard deviation of 0.31 %. These percentage values underscore MOSMA's robust and reliable performance in covering the solution space, making it a favorable choice over NSGA and MOPSO for multi-objective optimization tasks.

### 5.6 comparison with other published papers

Within this section, the implemented approach focuses on determining the optimal location and size for a constrained number of PV systems. The algorithm designed for this purpose is then simulated and applied to an IEEE 33 bus distribution network, featuring total active power and reactive demands of 3715 kW and 2300 kVAr, respectively. Various scenarios are considered, involving groups of 2 and 4 PV systems. The resulting locations and sizes derived from the proposed algorithm, along with comparisons to other methods, are presented in Table 6.

**Table 6:** A comparative analysis of the test results between the proposed algorithm and other existing methods.

Test case	Method	PV system size (MW)	Mean Voltage (pu)	Power loss (MW)
2 PV systems	MOSMA	0.9823, 1.0231	0.98931	0.07945
	Jaya [45]	0.84638, 1.15867	0.98039	0.08591
	Jamil [45]	2.138, 0.495	0.97871	0.09349
	NSGA-II [45]	0.5180, 0.4224	0.96606	0.10420
4 PV systems	MOSMA	0.5683, 0.8573, 0.9573, 0.7842	0.98960	0.05847
	Jaya [45]	0.58573, 0.83915, 0.98414, 0.68081	0.98344	0.06679
	Jamil [45]	1.54, 0.43, 0.376, 0.287	0.97776	0.08938
	NSGA-II [45]	0.9178, 0.4688, 0.8602, 0.8361	0.98490	0.06800

Table 6 presents a comparative analysis of the performance of the proposed MOSMA algorithm against other existing methods in scenarios involving 2 and 4 PV systems. For 2 PV systems, MOSMA outperforms Jaya, Jamil, and NSGA-II in terms of PV system size, mean voltage, and power loss. MOSMA achieves a PV system size improvement ranging from 13.27 % to 22.12 % over Jaya, highlighting its efficiency. Similarly, significant improvements are observed in mean voltage and power loss compared to Jaya, Jamil, and NSGA-II. In the case of 4 PV systems, MOSMA maintains its superior performance. It exhibits a PV system size improvement ranging from 7.21 % to 19.67 % over Jaya and 34.29 %–154.65 % over Jamil, showcasing its adaptability to more complex scenarios. Moreover, MOSMA demonstrates improvements in mean voltage and power loss compared to Jaya, Jamil, and NSGA-II. These percentage-based comparisons accentuate MOSMA's consistent and substantial advantages over alternative algorithms across different test cases, reinforcing its potential for effective optimization in distribution systems with varying numbers of PV systems.

## 6 Conclusion

In summary, the proposed multi-objective SMA method successfully optimized the allocation of photovoltaic resources in a 33-bus distribution network. Simulation results showcased its effectiveness in reducing losses and enhancing the voltage profile. For scenarios involving two or three photovoltaic sources, specific buses and capacities were selected, demonstrating improved voltage profiles and minimized losses. The analysis of varying penetration levels highlighted potential overvoltage issues in specific buses at higher penetration levels. Optimal positioning and capacity determination using the SMA algorithm addressed these challenges, suggesting that higher capacities should be installed at the initial buses for improved voltage profiles. The method's robustness was evident, considering its ability to handle objectives such as minimizing losses, improving voltage profiles, and achieving desired penetration levels. Valuable insights derived from these simulations offer practical guidance for decision-making in deploying photovoltaic systems within distribution networks.

One valuable future suggestion is to integrate energy storage systems into the optimization process. This allows for better utilization of renewable energy sources, improved grid stability, peak shaving, time-shifting of energy, and cost optimization. By considering the optimal sizing, placement, and operation strategies for photovoltaic systems and energy storage, the integration of storage technologies can enhance the effectiveness and efficiency of photovoltaic resource allocation.

## Declaration

The authors declare that there is no conflict of interest arising from this work.

## Data availability statement

Data included in article/supp. material/referenced in article.

## CRediT authorship contribution statement

**Zebin Wang:** Writing – review & editing, Writing – original draft, Formal analysis, Conceptualization. **Yu Li:** Writing – review & editing, Writing – original draft, Software, Methodology, Investigation, Formal analysis, Data curation, Conceptualization. **Guodao Zhang:** Writing – review & editing, Writing – original draft, Validation, Software, Methodology, Formal analysis. **Xiaotian Pan:** Writing – review & editing, Writing – original draft, Software, Methodology, Formal analysis, Data curation, Conceptualization. **Ji Li:** Software, Methodology, Formal analysis, Conceptualization.

## Declaration of competing interest

The authors declare that they have no known competing financial interests or personal relationships that could have appeared to influence the work reported in this paper.

## Figures and Tables

**Table A:** information of 33 bus distribution network [47].

Bus no.	From	To	resistance (ohm)	reactance (ohm)	active load power (kW)	reactive load power (kVar)
1	1	2	0.0922	0.0477	100	60
2	2	3	0.493	0.2511	90	40
3	3	4	0.3660	0.1864	120	80
4	4	5	0.3811	0.1941	60	30
5	5	6	0.8190	0.7070	60	20
6	6	7	0.1872	0.6188	200	100
7	7	8	0.7114	0.6188	200	100
8	8	9	0.030	0.74	60	20
9	9	10	1.04	0.74	60	20
10	10	11	0.1966	0.0650	45	30
11	11	12	0.3744	0.1238	60	35
12	12	13	0.4680	1.155	60	35
13	13	14	0.5416	0.7129	120	80
14	14	15	0.5910	0.5260	60	10
15	15	16	0.7463	0.5450	60	20
16	16	17	1.289	1.721	60	20
17	17	18	0.732	0.574	90	40
18	2	19	0.1640	0.1565	90	40
19	19	20	1.504	1.3554	90	40
20	20	21	0.4095	0.4784	90	40
21	21	22	0.7089	0.9373	90	40
22	3	23	0.4512	0.3083	90	50
23	23	24	0.8980	0.7091	420	200
24	24	25	0.897	0.7011	420	200
25	6	26	0.2030	0.1034	60	25
26	26	27	0.2842	0.1447	60	25
27	27	28	1.059	0.9337	60	25
28	28	29	0.8042	0.7006	120	70
29	29	30	0.5075	0.2584	200	600
30	30	31	0.9744	0.9630	150	70
31	31	32	0.3150	0.3619	210	100
32	32	33	0.3410	0.5302	60	40
